# Computational models of adaptive behavior and prefrontal cortex

**DOI:** 10.1038/s41386-021-01123-1

**Published:** 2021-08-13

**Authors:** Alireza Soltani, Etienne Koechlin

**Affiliations:** 1 Department of Psychological and Brain Sciences, Dartmouth College, Hanover, NH USA; 2 Institut National de la Sante et de la Recherche Medicale, Universite Pierre et Marie Curie, Ecole Normale Superieure, Paris, France

**Keywords:** Cognitive control, Human behaviour, Cortex

## Abstract

The real world is uncertain, and while ever changing, it constantly presents itself in terms of new sets of behavioral options. To attain the flexibility required to tackle these challenges successfully, most mammalian brains are equipped with certain computational abilities that rely on the prefrontal cortex (PFC). By examining learning in terms of internal models associating stimuli, actions, and outcomes, we argue here that adaptive behavior relies on specific interactions between multiple systems including: (1) selective models learning stimulus–action associations through rewards; (2) predictive models learning stimulus- and/or action–outcome associations through statistical inferences anticipating behavioral outcomes; and (3) contextual models learning external cues associated with latent states of the environment. Critically, the PFC combines these internal models by forming task sets to drive behavior and, moreover, constantly evaluates the reliability of actor task sets in predicting external contingencies to switch between task sets or create new ones. We review different models of adaptive behavior to demonstrate how their components map onto this unifying framework and specific PFC regions. Finally, we discuss how our framework may help to better understand the neural computations and the cognitive architecture of PFC regions guiding adaptive behavior.

## Introduction

Humans and other animals have evolved in a world that is uncertain, ever changing, and constantly presents choice situations that have been seen before rarely. These characteristics of natural environments—uncertainty, non-stationarity (volatility), and open-endedness—pose critical adaptive challenges, which ultimately determine the animal’s ability to learn about sources of food and danger and to take appropriate actions. To successfully tackle these challenges, animals must adapt their learning and decision-making strategies in multiple ways. Mammals are the most adaptive class of species as evident from their success in populating very different environments on the planet. The ability to adapt to different environments mainly relies on the brain cognitive flexibility, and not surprisingly, mammalian brains have evolved in specific ways related to the demand for adaptability [1] (also see [2] for more detailed discussion [R3.6]). Notably, the prefrontal cortex (PFC) has extensively evolved in mammals and especially in humans, suggesting the importance of the PFC for adaptive behavior.

Here, we aim to present a unified framework for understanding adaptive behavior in terms of different learning strategies that link stimuli, actions, and outcomes to guide behavior. In this framework, adaptability arises from specific interactions between multiple learning systems––each implementing different strategies––that are combined into task sets driving behavior. We propose that the main role of the PFC in adaptive behavior is to manage the learning and selection of task sets based on their reliability in predicting external contingencies, i.e., stimulus–action–outcome contingencies. We review existing computational models of adaptive learning and decision making to show how these models can be mapped onto different components identified in our framework. After establishing the link between existing models and our framework, we discuss contributions of different areas and regions of the PFC to adaptive learning and decision making. We then describe how our framework may help future research to better understand adaptive learning and decision making in the PFC by mapping computations that are currently considered as disparate processes to a unified machinery subserving adaptive behavior.

## Evidence for adaptability in learning and decision making

While an animal is interacting with its environment, not only does the environment change in multiple ways but also the animal’s internal state (e.g., needs) changes constantly. Each type of change requires specific adjustments in learning and decision making [3]. In this section, we provide examples of such changes and evidence for corresponding adjustments measured in controlled experimental settings.

### Naturalistic challenges that necessitate flexibility

Environmental changes include but are not limited to alterations in external contingencies in terms of the rate or probability at which different stimuli, actions provide reward, resulting in uncertainty [2, 3]; how reward can be obtained (model of the environment); and occurrences of new situations featuring new possible stimuli, actions, outcomes, and contingencies across these events. For example, food or water sources can be replenished at different rates during a season, requiring an animal relying on them to adjust the time or frequency at which it visits those sources. However, seasonal changes can drain some of those sources, forcing the animal to look for new ones. In search of new sources, the animal is faced with new landscapes and landmarks with different levels of risks regarding resource scarcity and predation. In addition to these external factors, as the animal’s physiological or motivational states change due to depletion or repletion, the desirability or subjective value of certain rewards may change. For example, as a thirsty animal drinks water from a waterhole, the reward value associated with that waterhole as a stimulus (often referred to as stimulus value should increase, whereas the value of drinking water as an action (often referred to as action value) should decrease, allowing the animal to attend to other needs and actions without reducing the predictive value of the waterhole in providing water (i.e., dissociate “objective” prediction of an outcome from its subjective value). Finally, contextual cues such as the presence of other non-predatory animals can provide additional information about the reward predictive value of certain stimuli or actions.

The aforementioned examples highlight important learning and decision-making challenges that animals face in natural environments featuring uncertain, volatile, and open-ended situations. In general, these features require simultaneous updates of different models that the animal uses to link stimuli, actions, reward outcomes (which we refer to as internal models), as well as adjustments in how information from these internal models should be combined to make choices. However, updating internal models that contribute to ongoing behavior should differ from previously learned models that do not. Non-stationary or volatile environments require adjustments in learning and/or in weighting different estimates from various learning systems. Finally, open-ended environments necessitate to regulate the generalization and transfer of previously learned models to novel situations and tasks.

To understand how the brain resolves these adaptive requirements arising in natural environments, similar situations are recreated in controlled experimental settings. These settings provide evidence that humans and other mammals exhibit different types of flexibility required to tackle them.

### Effects of uncertainty on learning

The probabilistic reversal learning (PRL) task and its variants have often been used to study the effects of uncertainty and volatility on learning and decision making [3], pointing to multiple types of behavioral adjustments. Using the PRL task with different reward probabilities for the better and worse stimuli, Costa et al. [4] found that to detect reversals in stimulus–outcome associations, monkeys rely more heavily on what they have learned (priors) in more uncertain environments (reward probabilities closer to 0.5), pointing to adjustments in inference processes to detect reversals according to expected uncertainty. Similarly, Grossman et al. [5] have shown that a model with the learning rate (i.e., the rate at which reward estimates are updated) that can increase or decrease depending on unexpected uncertainty (computed using unsigned reward prediction error) can better capture choice behavior of mice during a dynamic foraging task.

Other studies have found higher learning rates in volatile compared to stable environments in both monkeys [6] and humans [7, 8]. More detailed analyses and modeling of reversal learning, however, have provided evidence for time-dependent adjustments in learning relative to the time of reversals in monkeys [9] and humans [10].

### Effects of uncertainty on combination of information

Uncertainty and volatility have other profound effects beyond changes in learning rates. For example, a recent study examining learning and choice behavior across different experiments in monkeys and humans has found that reward probability and magnitude are combined in an additive fashion (instead of a multiplicative model based on the normative account) under uncertainty, and the relative weighting of reward probability to magnitude depends on the level of volatility in the environment [11]. In a similar task, Blain and Rutledge [8] also found that an additive model explains the combination of reward information in humans better than a multiplicative model. Interestingly, even when reward probability and magnitude are explicitly given but risk pressure changes over time, human subjects combine this information additively [12]. Consistent with these results, Rouault et al. [13] also showed that instead of optimal integration of reward magnitudes and belief about reward contingencies, human participants additively combine context-dependent reward expectations and reward magnitudes to make decisions under uncertainty. Moreover, using a probabilistic learning task in which monkeys had to learn the probability of reward for three stimuli, Wittmann et al. [14] showed that recent memories of unassociated reward and choice outcomes influenced future choices. Finally, there is evidence that adaptive behavior in volatile and open-ended environments is likely achieved through approximate low-level inferential processes about the *current* latent state of environment (that especially determines action–outcome contingencies) without inferring possible higher-order causes of changes in the environment such as the level of volatility [15].

### Adjustments in predicting reward

In addition to the probabilistic nature of reward outcome and changes in reward contingencies, an important form of uncertainty in the environment is the nature of stimulus–action–outcome associations or simply what predicts reward outcomes. This is especially challenging in natural environments because stimuli predicting reward outcomes have multiple features and are presented simultaneously, and thus, it is unclear what feature(s), combination(s) of features, and/or stimulus (stimuli) reliably predict reward outcomes and must be learned. Moreover, there are different ways that stimuli and actions preceding an outcome could be linked together. On the one hand, reward magnitudes can be used to associate presented stimuli and the chosen actions to estimate the so-called “cached values” for taking actions based on stimuli, which is usually referred to as model-free reinforcement learning (RL) [16]. On the other hand, actions (or sequences of actions) can be directly linked to outcome identity in order to allow predicting outcomes based on stimuli and states, leading to what is usually referred to as model-based RL [16].

There is evidence that these two types of RL are involved in a flexible manner. For example, in a two-step task in humans [17], the relative involvement of model-free and model-based RL appears to depend upon the prediction precisions associated with these processes [18] along with cognitive demands due to a concurrent task [19]. A more recent study in mice has found that volatile transitions across successive behavioral steps slows down the adaptive response to changes in reward probabilities and moreover, drastically changes how previous outcomes and interactions between transitions and outcomes influence choice behavior [20]. Finally, when facing multi-dimensional stimuli, there is uncertainty about which stimulus features should be learned to guide behavior to optimize adaptive behavior. For example, one can associate reward to individual features of stimuli and combine this information to estimate values associated with each stimulus (feature-based learning) instead of directly learning the value of individual stimuli (object-based learning). Recent studies showed that in response to multi-dimensional stimuli, the learning strategy also depends on the volatility, generalizability (i.e., how well features of stimuli or options predict their values), and dimensionality of the environment [21–23].

Together, these studies provide evidence that various mechanisms are involved in learning and decision making to guide adaptive behavior.

## A unifying framework for understanding adaptive behavior

**Fig. 1:**
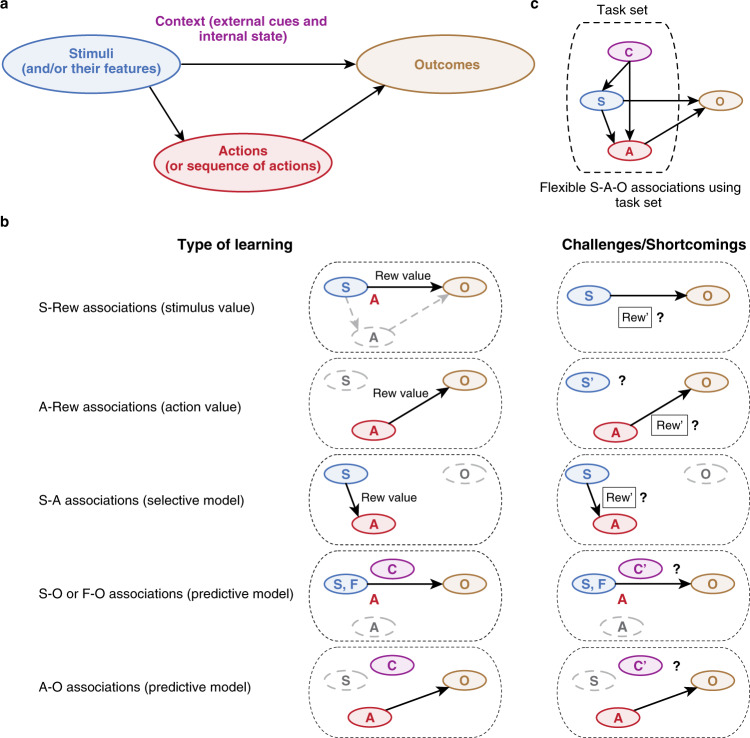
Dissecting adaptive behavior based on different types of links between stimuli, actions, and outcomes. **A** The goal of learning is to obtain certain outcomes by selecting appropriate actions based on presented stimuli while considering the context that includes internal state as well as external cues that reflect the latent state of the environment. This requires linking stimuli, actions, and outcomes, which can be done in multiple ways each with different levels of flexibility. **B** Different types of learning strategies for linking stimuli (S), actions (A), and outcomes (O) and their main shortcomings. (1) S-Rew associations link reward values (Rew value) of the outcomes to certain stimuli that precede those outcomes, allowing for the computation of stimulus value. Such a model cannot correctly link S and O if reward that follows the same stimulus (Rew’) or the state of the animal changes. (2) A-Rew associations link reward values (Rew value) of the outcomes to certain actions that precede those outcomes, allowing for the computation of action value. Such a model cannot correctly link A and O if reward (Rew’) that follows the same action or state of the animal changes. (3) S–A associations or selective models link the chosen action and the stimulus that precedes this action using experienced rewards. Such models cannot link S and A if reward type or state of the animal changes. (4) S–O (similarly feature–outcome, F–O) associations or predictive models link S (respectively, F) and O by learning the probability of outcomes contingent upon stimuli and/or their features regardless of their rewarding values through encoding the statistical occurrences of these outcomes. (5) A–O associations or predictive models link A and O by learning the probability of outcomes contingent upon actions regardless of their rewarding values. Predictive models cannot easily transfer learning from one context to another context. **C** Flexible link between stimuli, actions, and outcomes through creation of task sets consisting of multiple internal models (see text for more details).

The ultimate goal of learning is to enable the animal to exhibit an appropriate response or select an action with desirable outcomes in every situation based on presented stimuli, context, and the state of the animal (Fig. 1). Learning involves multiple internal representations with various degrees of flexibility. In its simplest form, learning leads to associate rewards (understood here and thereafter in terms of their subjective values) to certain stimuli and actions that precede them. Stimuli and actions thus acquire a rewarding value by being directly associated with rewards or indirectly via secondary reinforcers (like numbers or dots representing monetary values or reward quantities) through Pavlovian and simple instrumental conditioning, respectively. Learning stimulus-reward (S-Rew) and action-reward (A-Rew) associations (referred to as stimulus and action values, respectively) enables stimuli and actions to directly elicit desirable behavior. These types of learnings, however, do not form any internal models of the environment, as the learning primary reflect the current animal’s state or need (but see [24]). As a result, S-Rew and A-Rew learning are poorly flexible because stimulus or action values are divorced from outcome identity (e.g., make no differences between water or food rewards), hindering their use and integration when the animal’s state or need changes or when multiple types of reward are present.

### Selective models

Instead of associating reward to stimuli or actions that precede them (S-Rew and A-Rew learning), the brain can use reward to directly link the chosen action and the stimulus or stimuli that precede it (i.e., stimulus–action associations, S–A). In response to stimuli, the most desirable action can then be selected by choosing the action with the strongest stimulus–action association. In the context of habitual vs. goal-directed behavior, similar types of associations are referred to stimulus–response associations and attributed to the habit system [25]. However, we suggest that S–A associations form an internal model of the environment reflecting the reward magnitude contingencies upon the actions chosen in response to stimuli, which we refer to as a *selective model* [26, 27]. In our view, selective models are internal models that can be invoked beyond the notion of habits or model-free RL to covertly sample or replay additional internal models of the environment in the context of goal-directed behavior (see below).

Selective models improve adaptive behavior beyond S-Rew and A-Rew learning because they allow selecting actions according to stimuli, and because they can be used in conjunction with other internal models as explained below. Learning selective models can be simply performed through model-free RL based on Hebbian plasticity modulated by rewards. However, efficient RL requires the speed of learning (or learning rates) in selective models to depend in a complex manner upon the environment properties. Moreover, learning S–A associations can be challenging in natural environments featuring a myriad of stimuli or choice options, yielding to the curse of dimensionality (i.e., learning challenge due to an exponential increase in the number of possible stimuli/options when stimuli/options have multiple features each with multiple values) and credit assignment (i.e., correctly assigning outcomes to stimuli that predict them or to actions that resulted in obtaining them) issues. Finally, similar to S-Rew and A-Rew learning, selective models are poorly flexible again because S–A associations are divorced from outcome identity, hindering their use and integration when animal’s internal states and needs possibly in relation with the context (e.g., presence of predators and dangers) change.

### Predictive models

These adaptive issues can be overcome by learning the probability of outcomes contingent upon stimuli and/or actions regardless of their rewarding values and by encoding the statistical occurrences of these outcomes in stimulus–outcome (S–O), action–outcome (A–O) or stimulus–action–outcome (S–A–O) associations. These S–O and (S–)A–O associations form internal models of the environment that aim to predict outcome identity based on presented stimuli and/or actions, which we refer to as *predictive models*. Predictive models enable the brain to select actions based on anticipating outcome identity (directly from (S–)A–O associations or indirectly from S–O associations by remapping stimulus onto action spaces) and consequently based on *covertly experiencing* (i.e., without any action taken or reward being obtained) their current rewarding values. Predictive models are similar to cognitive maps [28] and form the basis for the so-called goal-directed behavior [25] but as explained below, serves an additional essential purpose, namely the arbitration between different sets of internal models to drive behavior.

Moreover, for simplicity, we use O to refer to outcome that may consist of many sensory features O_i_. Predictive models thus serve to predict O, O_i_, or combinations of O_i_’s occurrences. Using O as a generic notation, prediction is indeed about whether O will occur or not, regardless of its subjective values. If O varies on a magnitude/graded scale, then predicted models code the prediction as a certain level of O or about not less/more than a certain level of O. Finally, S and in S-Rew can also be encoded as O in predictive models. Such stimulus values S-Rew may compete in decision making with the anticipation of O based on predictive models and consequently the associated covert experience of the current subjective value of O. We note that this covert experience might then gradually alter the acquired S-Rew values internally. However, because this only can happen gradually, S-Rew values remain more rigid than current value anticipation processes based on predictive models solely.

Therefore, predictive models yield to more flexible behavior that depend upon animal’s internal states. In natural environments when stimuli have multiple features, predictive models can also learn to associate outcomes to stimuli features (F–O associations), thereby mitigating the curse of dimensionality [21–23]. In addition, predictive models can be extended to include multiple stimulus, actions, and outcome and thus can incorporate the true interdependence between stimuli, actions, and outcomes in the environment. This makes predictive models more precise than selective models reflecting only reward magnitudes. At the same time, predictive models are learned through co-registration of stimuli or stimulus’ features, actions, and outcomes, making them more complex to learn than selective models especially in the presence of other (distractive) events.

### Advantages and disadvantages of selective vs. predictive models

Both selective and predictive models enable the animal to choose desirable courses of action. They have their own advantages and disadvantages, yielding to the important question regarding how they are combined to make decisions or equivalently, how their respective influence on choice is regulated. Selective models directly incorporate rewarding/punishing subjective values when learning occurs making them especially efficient in the short run or when such values are weakly dependent upon animal’s internal states but rather inefficient in the converse cases. Predictive models, by contrast, incorporate more extensive and “objective” information about the environment, making them slower to learn and less efficient in the short run but much more efficient to guide behavior with respect to the animal’s internal states that may vary across time.

The contribution of selective and predictive models to adaptive behavior was inspired by similar interactions between model-free and model-based RL systems [16, 18]. However, unlike pure competition between model-free and model-based RL in controlling behavior and arbitration between the two systems based on their reliabilities, the respective contribution of selective and predictive models to adaptive behavior should be viewed as being cooperative and mutually supportive, as originally proposed in Artificial Intelligence [29]. Indeed, selective and predictive models can learn external contingencies in parallel because predictive models can serve as an internal model to covertly (i.e., without taking any action) simulate the environment, which selective models may covertly sample and consequently learn with respect to current animal’s internal states. Accordingly, adaptive behavior is likely to derive from a constant mixture of signals from selective and predictive models invoked together [30], which weights should especially rely on the reliability of outcome expectations drawn from predictive models [27, 29, 31–33].

### Shortcomings of selective and predictive models and how they can be mitigated

A central challenge for adaptive behavior, however, is that in the learning mechanisms mentioned above, new associations are learned when new situations occur by gradually erasing previously learned associations. Although this has little impact when previously learned situations never reoccur, overwriting learned associations is dramatically detrimental in open-ended environments featuring both recurrent and new situations [26, 27]. Therefore, additional adaptive mechanisms are needed to deal with recurrent and new situations in order that learning new associations preserves what was previously learned and that previously learned associations may be retrieved to guide behavior.

We have previously proposed that this requirement for efficient adaptive behavior in uncertain, non-stationary, and open-ended environments is achieved by creating task sets––that is, combinations of selective and predictive models along with contextual models (see below)––that are invoked and stored together in relation with latent states of the environment to efficiently adapt to both recurrent and new situations [26, 27, 34]. In our framework, task sets correspond to large-scale neural frames (i.e., combinations of interacting neural activity across multiple brain regions) linking together several internal (selective, predictive, and contextual) models encoded in multiple brain regions to be invoked together to drive behavior when the corresponding hidden state occurs. Therefore, task sets are instantiations of external latent (hidden) states similar to the notion of task state [35].

### Unified framework and its computational components

Considering these adaptive challenges altogether with regard to uncertain, non-stationary, and open-ended environments, we extend here our previous framework [26, 27, 34] to propose that adaptive behavior derives from the following computations, not all of which are present in all mammals. Importantly, we suggest that although most of these computations involve multiple cortical and subcortical regions outside the PFC, the critical role of the PFC is to combine internal models into task sets and to arbitrate between task sets that allow ultimate flexibility as explained below:

1. Simultaneous learning of a hierarchy of selective models spanning multiple timescales within the same task set. Timescales refers to the time decay in the influence of previous rewards on learned associations [36], whereas temporally distant events (stimuli, actions) can be linked to rewards via eligibility traces (i.e., variables used to track past events over time to potentially associate them with temporally distant subsequent events) occurring at various timescales [37].
2. Simultaneous learning of a hierarchy of predictive models spanning multiple timescales and complexity levels within the same task set. Complexity levels notably reflect the combinatorial complexity of associations between stimuli or features of stimuli, actions, and outcomes (S–A–O, S–O, A–O associations), resulting in different learning strategies across these levels.
3. Inferring latent states in the environment by monitoring the reliability of outcome expectations from the various predictive models within the actor task set–– that is, the task set guiding ongoing behavior––to allow changing the actor task set for a new task set to guide subsequent behavior. Indeed, an actor task set with reliable predictive models (i.e., more likely matching than not matching current external contingencies) that become unreliable indicates a change in the current latent state of the environment [26, 34]. This inferred change leads to create a new actor with new internal models retrieved from long-term memory while preserving the old task set in long-term memory for future retrieval and use.
4. Learning an additional type of internal models, named *contextual models* that learns on the one hand external cues predicting rewards associated with selective models and external cues predicting the reliability of predictive models on the other hand. As we previously proposed [26, 27, 34], contextual models enable to build new actor task sets as appropriate actor priors from properly mixing task sets stored in long-term memory when their associated latent states re-occur. When conversely the animal faces an entirely new situation (latent state) it never experienced before, the new task set simply results from an uninformative mixture of previously stored task sets as a proper actor prior aiming at learning new external contingencies. Overall, task set reliability that governs the maintenance and creation of actor task sets reflect the overall reliability of task set’s predictive models, i.e., the reliability based on the likelihood of outcomes and contextual cues given predictive and contextual models, respectively. Such reliability inferences may be computed through forward Bayesian inferences or through inference proxies involving these internal models (see below).
5. Utilizing and adjusting the internal models within the actor task set. In this framework, only the internal models within the actor task set drives ongoing behavior and are updated accordingly. This implies that only certain learning strategies and accompanying representations could remain active (e.g., in working memory) and thus are updated. For example, representations of only a subset of stimuli could remain active or only a subset of recent stimuli and their features (or combination of features), states, and actions remain active, allowing updates in a small number of selective and predictive models, respectively. Selecting or forming an actor task set, a process which might reflect selective attention, effectively prunes the repertoire of possible models to avoid an exponential increase in their number. Within the actor task set, both selective and predictive models contribute to behavior according to the reliability of predictive models. The more predictive models are reliable, the more they contribute to behavior, and vice versa. This reliability is computed proactively from contextual models given contextual cues and reactively from the predictive models given actual action outcomes.
6. Storing task sets in long-term memory as large-scale neural frames linking together task sets’ internal models. This storage enables to create new actor task sets as mixtures of stored task sets weighted by their contextual models given contextual cues to retrieve the relevant internal models for rapid adaptation to recurrent situations (i.e., recurrent latent states) or to transfer this knowledge as proper priors to adapt to new situations (i.e., new latent states).

These computations and processes extend our previous framework [26, 27] in multiple ways. First, here we consider that task sets also comprise additional predictive models based on S–O associations divorced from actions and directly predicting outcomes from stimuli or their features. Such models may also contribute to behavior by remapping stimuli or their features onto action spaces.

Second, considering the combinatorial and temporal extent of external contingencies in natural environments, we propose that the animal updates hierarchies of selective and predictive models (within the actor task set) at various timescales and different complexity levels, consistent with the extensive heterogeneity of neural responses to rewards and outcomes across the cortex [38]. Although presumably, only a small part of these internal models contributes to behavior at one time, learning this variety of internal models enables the brain to subsequently create more efficient multi-scale task sets and to better estimate their reliability.

Third, adaptive behavior derives from either adjusting the actor’s internal models while perseverating with the same actor or switching to a new actor for guiding subsequent behavior. Arbitration between these two alternatives is based on inferring the actor reliability. Here we further propose that the actor reliability integrates the reliability of multiple predictive models, which are evaluated separately to determine their contribution to behavior. A task set is said reliable when collectively, the predictive models more likely match than do not match current external contingencies (see [26] for a proper computational definition). It is said unreliable in the converse case.

Fourth, we further suggest that within task sets, contextual models comprise internal models that learn external cues predicting the rewards associated with distinct selective models and internal models that learn the reliability of predictive models, allowing to proactively weight their relative contribution to behavior before experiencing action outcomes.

Fifth, we assume that besides predictive models comprising S–A–O associations, task sets include predictive models comprising S–O and A–O associations which number grow much slower than S–A–O associations Therefore, even though more complex models are more accurate in estimating and predicting external contingencies, these simpler models are faster to learn, as being more generic and less precise than more complex models that require experiencing more specific combinations between stimuli, actions and outcomes. Consequently, simpler models may enable faster adaptations to more volatile environments, whereas more complex models could exhibit biases when the environment changes quickly.

Finally, all computations outlined above are unlikely to be observed in all mammals. As previously argued based on anatomical evolutionary considerations [26], the learning of contextual models that enables proactive reliability inferences might have evolved only in higher mammals such as primates with the evolution of the mid-lateral prefrontal cortex. In humans, additionally, the evolution of the lateral frontopolar cortex is viewed as endowing humans with the ability to jointly monitor the reliability of several task sets in parallel, namely the actor task set along with two/three additional task sets [26, 39]. These additional task sets are named counterfactual because they are neither contributors to ongoing behavior nor updated accordingly. However, they endow humans with two new key adaptive capabilities: (1) switching reversibly between concurrent actors according to their respective reliability without resorting on the notion of task set creation; and (2) transforming the notion of task set creation into hypothesis-testing: when no monitored task sets (including the actor) are deemed reliable, a new task set is tentatively created to serve as actor as described above and monitored along the others. This “probe” actor is generally unreliable initially but by learning from experience, will eventually become reliable. However, this new task set may be subsequently disbanded when it remains unreliable while one counterfactual task set become reliable again and consequently selected as actor. These two capabilities yield to the notion of directed exploration and perhaps to uniquely human reasoning abilities in the service of more flexible adaptive behavior [27].

### Relationship to other frameworks

Considering parallels between selective vs. predictive models and concepts of habitual vs. goal-directed systems [25, 40–43] or model-free vs. model-based RL [16, 18], we would like to highlight similarities and differences between these and our frameworks in understanding adaptive behavior (also see chapters by Collins and Shenhav [44], and by Averbeck and O’Doherty [45] in this special issue).

First, similar to habitual vs. goal-directed and model-free vs. model-based RL dichotomies, selective and predictive models are more concerned with generating a response and predicting the outcome, respectively. Moreover, learning in habitual vs. goal-directed systems or model-free vs. model-based systems is often assumed to be independent of each other while each of the two systems compete with each other to control behavior based on their reliabilities. Some also have suggested that interactions between the two systems could be hierarchical such that goal-directed behavior can activate habitual mechanisms [46] or vice versa [47]. In contrast, interactions between selective and predictive models are more cooperative because while selective and predictive models learn external contingencies in parallel and compete for action selection within the actor task set, predictive models can serve as an internal model to simulate the environment which in turn is sampled by the selective models to learn according to current animal’s internal states. That is, the two types of models can use other models to learn more efficiently.

More importantly, in contrast to arbitration between habitual vs. goal-directed systems or between model-free vs. model-based RL, we suggest that arbitration occurs between task sets; i.e., either perseverating with the same actor task set (and consequently relying on and adjusting the same internal models to drive behavior) or switching to another actor task set (and consequently relying on other internal models to drive behavior). In particular, this task-set arbitration may result in activating selective models from the new actor task set to drive behavior based on (the unreliability of) predictive models within the old actor task set, a process that can appear as the so-called mixture of habit and goal-directed behavior or model-free and model-based RL.

Our unifying framework provides a systemic view and a systematic way to study adaptive behavior in terms of underlying computations that can be applied across experimental designs and tasks (Fig. 1). Although some of the described processes are more difficult to identify in certain experimental paradigms and tasks, we suggest that all these processes are elicited and contribute to some extent to any adaptive behavior. Therefore, independently of tasks and experimental protocols, our framework provides a lens by which different learning processes, especially those implemented in the PFC can be viewed as parts of a unified machinery driving adaptive behavior.

## Computational models of adaptive learning and decision making

In this section, we provide an overview of recent computational models of learning and decision making and how these models can be mapped into our unifying framework. Our aim is not to provide an exhaustive review of existing models. Our main focus is to identify unique computational components underlying adaptive behavior and ultimately to map these components onto neural substrates. Because of the complementarity of selective and predictive models in guiding adaptive behavior, we categorize these computational accounts into those involving predictive models, those involving selective models, and those combining both.

### Computational accounts based on predictive models

Here, we examine computational models of adaptive learning and decision making based on predictive models. Learning predictive models naturally rely on probabilistic or Bayesian inferences, which are intrinsically related to learning external contingencies in uncertain environments [48].

Probabilistic inferences have been proposed to account for learning predictive models regarding current outcome contingencies and guiding decision making. In a stationary world, learning such contingencies consists of inferring the statistical regularities of outcomes given stimuli and/or actions. In non-stationary environments, however, learning such predictive models is much more complex, because the learning must take into account possible changes in latent states of the environment, i.e., the possible changes in external contingencies over time. To account for this adaptive challenge, several computational models involve a hierarchy of inference processes about latent states comprising: (1) first-order inferences forming beliefs about the current latent state; (2) second-order inferences about the environment *volatility*––that is, the probability of latent state changes––modulating first-order beliefs; and (3) even third-order inferences about changes in the environment volatility regulating second-order inferences [7, 49–51]. Despite the usefulness of these models in revealing computations involved in integration of reward outcomes in changing environments, these models raise a computational complexity issue [R1.1]. They rapidly become computationally intractable, which questions their biological plausibility.

Consistently, algorithmic approximations have been proposed that rely on more explicitly detecting change points in latent states [4, 52–54]. For example, Nassar et al. [10] reduce a Bayesian change-detection model to a model based on delta rules that adjusts the influence of new outcomes according to the uncertainty and probability of change points. There are also models inspired by Kalman filters that keep track of both the estimated state of the system (e.g., reward probability) and the variance of these estimates in order to tackle learning under volatility [55, 56]. Other models use probabilistic inferences to more directly estimate outcome contingencies and uncertainties about these estimates ([48, 55, 57, 58]. There are also models of adaptive behavior that rely only on first-order probabilistic inferences about current latent states but combines these inferences with other mechanisms to adjust learning and decision making. For example, Faraji et al. [59] have proposed a model monitoring *Bayesian surprise*, i.e., the discrepancy between outcome likelihoods derived from first-order beliefs and actual outcomes, along with a notion of belief commitment, to drive adaptive learning by minimize such confidence-corrected Bayesian surprise.

In all these models, first-order beliefs about current latent states derive from predictive models and as outlined above, correspond to the notion of reliability associated with predictive models that contribute to task set reliability. However, despite the proposed approximations of higher-order probabilistic inferences about volatility, these models remain based on a complex algorithmic machinery which biological plausibility is again questionable. Surprisingly, Findling et al. [15] have recently shown that higher-order inferences about volatility and consequently these algorithmic approximations are actually unnecessary for efficient learning in non-stationary environments. Specifically, they demonstrate that first-order inferences alone about latent states are sufficient to reach near-optimal adaptive behavior and best account for human adaptive performances, provided that these inferences undergo computational imprecisions consistent with the psychophysical Weber’s law [60]. The result thus provide evidence that learning predictive models within the actor task set that guides ongoing behavior and inferring their reliability to possibly change the actor task set require only imprecise computations confined to simple first-order inferences about current latent states.

Predictive models predict outcome identity and alone, are unable to orient behavior towards *desirable* outcomes. The previously described models generally circumvent this issue by considering only binary rewarding outcomes (i.e., reward vs. non-reward outcomes), that allows conflating the notion of outcome identity with desirability. In natural environments, however, rewards are rarely binary. Efficient adaptive behavior thus requires predictive models to be combined with other information as the magnitude of rewards, i.e., the rewarding values of outcomes or in our terminology, stimulus values (S-Rew). One possibility is that reward magnitudes are incorporated within predictive models, so that predictive models learn not only to predict outcome identity but also reward magnitudes through probabilistic inferences. The resulting predictive models however become highly complex to learn and to draw first-order inferences about latent states. Another possibility is that reward magnitudes are divorced from predictive models and consequently from outcome identity and are learned as stimulus and/or action values or as S–A associations composing selective models through RL. These considerations yield us to now review models of adaptive behavior based on selective models.

### Computational accounts based on selective models

Most early models of adaptive behavior focus on associating rewards to cues or actions that precede them. These models correspond to learning stimulus values (S-Rew), action values (A-Rew), and S–A associations composing selective models through model-free RL based on reward prediction errors [61, 62]. These models, however, remains too simple to face the adaptive challenges raised by natural environments including their volatility and open-endedness along with the multiplicity of stimuli accompanying rewards as revealed in downward unblocking paradigms. Tackling these challenges requires additional components as proposed in subsequent models. For example, competition between stimuli for learning [63] or competition during representation [64] can account for downward unblocking and allows for flexibility in the learning rate depending on the outcome unexpectedness.

Similarly, more recent models have used (unsigned) reward prediction errors to adjust the learning rate or to construct a dynamic learning rate [65–68]. Alternatively, the history of unsigned reward prediction errors has been used to estimate the expected uncertainty to scale the learning rate in a dynamic action-based learning task [5]. Moreover, in a recent model by Wittmann et al. [14], a conventional model-free RL model was supplemented with multiple components that track recent rewards and choices in terms of location and stimulus in order to capture the effects of choice and reward history on behavior during an armed bandit task with three stimuli. Importantly, these models are extensions of conventional model-free RLs learning action values (A-Rew) or selective models (S–A associations). We note however that they often relate to experiments involving only binary rewards, which prevent from really distinguishing the formation of true action values A-Rew or S–A associations composing selective models from (S–)A–O associations composing predictive models. Note however that even with binary rewards, the distinction can be made in sequential, multi-steps tasks because of the presence of intermediate action steps before obtaining rewards.

Learning adjustments to the environment volatility were also captured by mechanistic models that adapt to reward statistics with neither any probabilistic inferences about outcome identity nor assumptions about the environment contingencies [9, 69, 70]. For example, Farashahi and colleagues [9] have shown that a specific structure of synaptic plasticity associated with multiple meta-stable internal neural states capturing the reward history leads to learning adjustments to expected and unexpected uncertainty resulting in time- and option-dependent learning rates.

In natural environments, there is myriad of stimuli and choice options each with multiple features or attributes, making association of rewards to stimuli challenging. To tackle this challenge often referred to as the curse of dimensionality, more recent models include reinforcement learning about features of stimuli [21–23, 71–73]. This feature-based learning strategy, which allows estimating stimulus values based on their features, can mitigate the curse of dimensionality by reducing the learning about all possible stimuli (object-based learning) to a much smaller number of feature-reward associations (feature-based learning) [21, 71]. Interestingly, Farashahi et al. [21] show that feature-based and object-based learning models can interact more efficiently based on the reliability of signals in the two models rather than based on choice accuracy. Moreover, more complex learning strategies accompanying feature-based learning are required for more flexibility [23]. Similarly, learning S–O associations when multiple stimuli are presented can be achieved by predicting more rewarding stimuli based on presented cues without any explicit probabilistic inferences [74, 75].

Finally, it has been proposed that model-free RL can learn to arbitrate between selective models within actor task sets. For example, Duverne and Koechlin [76] show that distinct selective models can acquire reward values through RL that allow arbitrating between them. Eckstein and Collins [77] further propose a hierarchical RL consisting of learning distinct selective models composed of S–A associations along with learning “selective models of selective models” composed of associations between contextual cues and selective models in order to arbitrate between the subordinate selective models to drive adaptive behavior across changing contexts.

### Computational accounts combining predictive and selective models

As noted above, selective models (S–A associations) along with stimulus values (S–Rew), action values (A-Rew) learn the magnitude of rewards/punishments through RL more efficient than predictive models (S–O, A–O, S–A–O associations) that capture statistical regularities of outcomes. Conversely, predictive models adapt behavior to changes in the animals’ internal states more efficiently than selective models. Therefore, combining selective and predictive models are certainly required to elicit efficient adaptive behavior.

Collins and Koechlin [34] propose a model combining selective, predictive, and contextual models within task sets to drive adaptive behavior. This model assumes that within the actor task set, the selective model learns through model-free RL and selects actions, while predictive models learn to predict action outcomes from selected responses to stimuli. Contextual models learn external cues predicting proactively the reliability of predictive models. Task sets’ reliability is computed through first-order probabilistic inferences regarding the reliability of predictive models, proactively according to contextual cues and reactively according to actual outcomes. While the actor task set is deemed reliable, it guides behavior. Otherwise, if other task sets are monitored along with the actor task set and one of them is deemed reliable, this task set is selected to serve as actor. If no other monitored task sets are deemed reliable, a new task set is then created from the mixture of task sets stored in long-term memory to serve as a probe actor task set. This probe actor is generally unreliable initially but learns from experience and guide behavior as long as no other monitored task sets become reliable. In the case another monitored task set becomes reliable, the latter becomes the actor, yielding the probe actor to be either disbanded if still deemed unreliable or stored in long-term memory in the converse case. Collins and Koechlin [34] show that this model forms a forward algorithmic approximation of optimal but computational intractable adaptive processes based on Dirichlet Processes Mixtures. They further show that all these components including the concurrent reliability monitoring of three/four task sets are necessary to account for human adaptive performances. One limitation of this model, however, is that within the actor task set, action selection is confined to a unique selective model, while task set reliability derives from the reliability of a unique predictive model within each task set.

Doya et al. [78] have proposed a multiple model-based RL model, which in a way conceptualizes how different internal models within the actor task set may be weighted to drive behavior concurrently. Their model comprises multiple pairs of selective and predictive models, named controllers. Predictive models serve to infer the reliability of each controller (which they refer to as responsibility signals), which is used to: (1) determine the weighs or relative contribution of each selective model to action selection; and (2) modulate the updating of internal models across all controllers following action outcomes. The model assumes that all controllers are similar in nature but might be extended to controllers operating at different timescales as suggested above.

In both Collins & Koechlin’s and Doya et al.’s models, only selective models directly contribute to action selection within the actor task set. These models provide no accounts of how action selection may derive from both selective and predictive models. One extreme view is that either selective or predictive models contributes to action selection [18]. Alternatively, Rouault et al. [13] propose that action selection derives from additive (i.e., independent) contributions of stimulus/action values, selective, and predictive models after normalizing each of these contributions across behavioral options to make them commensurable. Although such independent contributions are normatively sub-optimal, this mechanism minimize the impact of learning imprecisions on decision making and was found to account for human adaptive performances in non-stationary environments. The model of Rouault et al. [13], however, leaves open the issue regarding how in this additive combination, the different contributions are weighted relative to each other. We propose here that relative to selective models, the contribution of predictive models to action selection within the actor task set are weighted according to their reliability in predicting outcome identity. The hypothesis is consistent with previous findings showing that the weights of predictive relative to selective models in action selection decrease following unexpected changes in external contingencies [79] and more globally, when the environment volatility increases [11, 80]. Both cases indeed impact negatively the reliability of predictive models.

## Contributions of prefrontal cortex to adaptive behavior

**Fig. 2:**
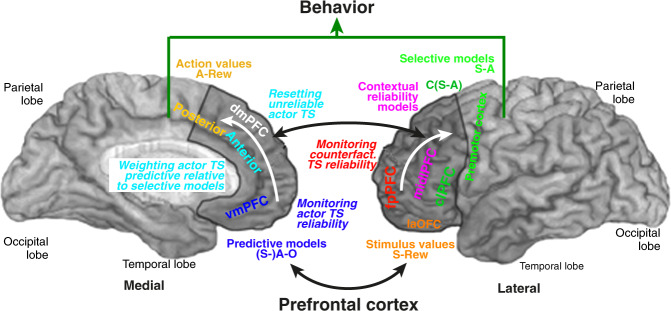
Functional architecture of the prefrontal cortex contributing to adaptive behavior. Medial and lateral view of the human prefrontal cortex (PFC) and its main anatomical regions shown in relation with their contribution to adaptive behavior. The laOFC (lateral orbitofrontal cortex), vmPFC (ventromedial PFC), dmPFC (posterior and anterior dorsomedial PFC) along with premotor cortex (and possibly clPFC) are present presumably in all mammals from rodents to primates and humans. The lateral PFC including clPFC and especially midlPFC (mid-lateral PFC) emerges in primates, whereas fpPFC (frontopolar cortex) is specific to humans. In the proposed framework, the laOFC encodes stimulus reward values (S-Rew), posterior dmPFC action reward values (A-Rew). The vmPFC encodes predictive models involving learning (Stimulus–)Action–Outcomes associations. The lateral premotor cortex encodes low-level selective models (Stimulus–Action associations), whereas the clPFC encodes higher-level selective models (Cues-(S–A) associations). Task sets (TS) form large-scale neural frames linking such internal models encoded in these various PFC regions in order to potentially invoke them together to guide behavior. TS reliability is the ability of TS internal models to jointly predict external contingencies. midlPFC learns contextual models predicting TS reliability according to external cues. The actor TS is the TS driving ongoing behavior and which reliability is monitored in the vmPFC. Counterfactual TS are the TSs which reliability are monitored in the fpPFC without contributing to ongoing behavior. White arrows indicate major information flows related to actor task set reliability (medial PFC) and counterfactual task-set reliabilities (lateral PFC). Black arrows indicate major information flows related to reward values of action outcome (ventral PFC) and reliability-based inhibition or selection of actor TS in the dorsal PFC. See text for more explanations.

In the previous sections, we proposed a framework to identify and integrate multiple computational mechanisms and components into a unified system driving adaptive behavior. In this section, we briefly review empirical findings indicating how these elements map onto the PFC (Fig. 2). While most of these computations also involve other brain regions (e.g., basal ganglia, hippocampus) that extensively interact with the PFC, we suggest that the PFC is more specifically devoted to form and select task sets driving behavior. We show how our unified framework provide an integrative view that may help to understand the functional organization of PFC.

### Selective models and the lateral premotor-prefrontal cortical complex

The premotor cortex is a transition area between the motor cortex and the PFC. There is extensive evidence in monkeys and humans that the lateral premotor cortex (lPM, Broadman’s Area 6, BA6) along with the adjacent, caudal sector of the lateral prefrontal cortex (clPFC), BA8, BA44/45), learns selective models that drive behavior. Monkey and human studies show that in relation with basal ganglia, the lPM learns S–A associations and selects actions associated with stimuli, whereas the clPFC learns hierarchically more complex selective models associating external cues to consistent sets of S–A associations; i.e., “selective models of selective models” driving context-dependent behavior (e.g., [81–90]).

Mechanistically, associations composing selective models and linking stimuli and actions according to reward values are likely stored at the synaptic level using activity-dependent synaptic plasticity modulated by reward signals [91]. Synaptic connections between neurons responding to presentation of a stimulus and neurons selective for an action can be potentiated or depressed depending on dopamine releases. This enables these synaptic connections to learn S–A associations and hierarchically more complex selective models, especially through the processing reward prediction errors in subcortical structures involved in RL (e.g., [92–94]). Stimulus values (S-Rew) or action values (A-Rew) are likely learned through similar RL mechanisms. The PFC receives massive dopaminergic projections and contain distinct regions with neurons encoding stimuli and/or actions, leading distinct regions to learn stimulus values and action values rather than selective models similar to many subcortical areas such as striatum that encodes both stimulus and action values [26].

### Stimulus values and the lateral orbitofrontal cortex

The orbitofrontal cortex (OFC) is present in all mammals and comprises a medial and lateral sector, with the latter (laOFC) receiving most sensory inputs to the PFC [95]. There is a long history of empirical evidence showing that the laOFC encodes stimulus values (S-Rew) divorced from actions in associations with subcortical structures [96, 97]. More recent studies notably using human neuroimaging confirm that the laOFC encodes stimulus values learned through RL [13, 98, 99]. Lesions of monkeys’ laOFC further impair S-Rew learning by altering correct assignment of rewards to chosen stimuli [100].

### Action values and the posterior dorsomedial prefrontal cortex

In contrast to the OFC, the posterior dorsomedial PFC (posterior dmPFC) including the presupplementary motor area and caudal parts of the anterior cingulate cortex is linked to the motor system. Although action values (A-Rew) divorced from stimulus are experimentally difficult to dissociate from stimulus values that elicit responses, human studies successfully dissociated the two value notions and found action values guiding selection to be encoded in the posterior dmPFC [101, 102]. The human posterior dmPFC was also found to encode the relative reward values between two distinct action sets (sets of sensorimotor associations) acquired through RL and guiding behavior [76], indicating that action values also bear upon action representations hierarchically higher than simple motor acts.

We note that many studies investigating learning and decision-making use binary rewards that conflate the notion of reward magnitudes and outcome identities. Except in sequential, multi-step tasks, the expectations about binary rewards confound reward magnitudes with outcome probabilities, making the notion of stimulus or action values (S-Rew or A-Rew) indistinguishable from the notion of predictive models comprising S–O and/or (S–)A–O associations. However, several studies that manipulates reward magnitudes independently of reward probabilities allow for identifying the neural bases of predictive models.

### Predictive models and ventromedial prefrontal cortex

The ventromedial PFC (vmPFC) refers to the prefrontal region including the medial OFC and the subgenual part of the anterior cingulate cortex. Studies across mammalian brains from rodents to monkeys and humans provide evidence that the vmPFC learns and encodes predictive models that predict action outcomes in response to stimulus (e.g., [13, 103–107]). For example, Hampton et al. [103] show that in humans, vmPFC activations in a probabilistic reversal learning task are more consistent with predicting reward probabilities than magnitudes. Boorman et al. [104] and Rouault et al. [13] also show that the human vmPFC encodes reward probabilities independently of reward magnitudes. Empirical evidence is that besides reward probabilities, the vmPFC also encodes the reward magnitudes expected from chosen actions (e.g., [13, 104]) and after the choice is made [108, 109]. This evidence suggests that once an action is chosen, the vmPFC uses predictive models to encode the likelihood of future action outcomes along with their rewarding values possibly encoded as stimulus values (S-Rew) in the adjacent laOFC.

Our framework points out the critical role of predictive models in inferring the actor task set reliability. A task set presumably form a large-scale neural frame linking together the collection of internal models involving distinct prefrontal regions as described above and that can be invoked together to learn and drive behavior in relation to a latent state of the environment. The actor task set is the one learning and driving ongoing behavior in the current latent state. While this task set remains reliable, the current latent state is unlikely to have changed and this task set is kept as actor. As hypothesized above, the task set reliability is inferred online and primary rely on monitoring the reliability of predictive models that compose it.

### Actor reliability and the vmPFC

We mentioned empirical evidence that the vmPFC encodes actor predictive models. Consistently, neuroimaging and intracranial EEG studies in humans provide explicit evidence that neural activity in the vmPFC further correlates online with the actor task set reliability inferred from predictive models given the occurrence of actual action outcomes [109, 110]. Actor reliability measures the probability that the current latent state of the environment remains unchanged. Other neuroimaging studies confirm that vmPFC activations are indeed associated with latent states determining current action outcome contingencies [111, 112]. Consistent with the idea of the OFC as a cognitive map of task space [35], we suggest that within task sets, the OFC is more specifically involved in encoding predictive models and additionally monitoring the reliability of the actor task set from predictive models for inferring whether the current hidden state has changed or not. This indeed gives a central role to the OFC in relation with latent or hidden states and point to the involvement of metacognition in estimating actor reliabilities. Indeed, actor reliability also reflects the confidence in perseverating with the same task set. Neuroimaging studies consistently show that subjects’ confidence judgments about their own performance are associated with vmPFC activations [113, 114].

The actor reliability signals observed in Donoso et al. [110] and Domenech et al. [109] simply reflect the reliability of actor predictive models. They might serve to weight the contribution of actor predictive relative to selective models to behavior. More radically, when the actor task set become unreliable, the current latent state of the environment has likely changed and as behaviorally observed, calls for a new actor task set [26, 34].

### From actor exploitation to exploration: the anterior dorsomedial PFC

The anterior dorsomedial PFC (anterior dmPFC) lies between the vmPFC and posterior dmPFC and particularly includes the dorsal anterior cingulate cortex (dACC). There is ample evidence across mammals that the neural activity in the anterior dmPFC reflect multiple value and reliability signals associated with actions, action–outcome, stimulus–action, and stimulus–action-outcome associations (e.g., [87, 115–119]) while lesions of ACC impair learning that relies on the integration of action outcomes over time [120], suggesting that the anterior dmPFC is involved in weighting the different contribution of actor internal models to guide adaptive behavior [87]. Supporting this hypothesis, Rouault et al. [13] found that in humans, the anterior dmPFC guides behavioral choices by collecting and weighting the independent contribution of learned stimulus values encoded in laOFC, beliefs about outcome probabilities (predictive models) encoded in the vmPFC, and reward magnitudes of expected outcomes encoded in the vmPFC. Schuck et al. [121] report evidence that relative to a default internal model guiding behavior, the anterior dmPFC increasingly weights another internal model that are gradually learned in parallel and that will guide subsequent behavior. Consistent with this integrative weighting function, the anterior dmPFC was found to combine pieces of behavior-relevant information over larger timescales than other prefrontal regions [38].

One may thus hypothesize that through the anterior dmPFC, actor predictive models contribute to behavior relative to selective models according to their reliability monitored in the adjacent vmPFC. In agreement with this hypothesis, Donoso et al. [110] and Domenech et al. [109] show that, anterior dmPFC neural activity in humans specifically responds when the actor predictive model and consequently the actor task set becomes unreliable, yielding to the creation of a new task set from long-term memory corresponding to the notion of undirected exploration. Rodent and monkey studies consistently show that abrupt phase transitions occur in the dACC neural ensembles in relation to behavioral switches [122–125]. Moreover, monkey electrophysiological and human neuroimaging studies indicate that the anterior dmPFC responds when unpredicted action outcomes trigger behavioral switches [106] and especially switches from exploitation to exploration behaviors [12, 126–128]. In particular, anterior dmPFC activations are observed when humans form new task sets to guide subsequent behavior [129]. Thus, the anterior dmPFC seems to play a pivotal role in weighting the different internal models within the actor task set and when the latter is deemed unreliable, in inhibiting this actor and eliciting new actor task sets to guide behavior.

All the prefrontal regions reviewed above are present in all mammals [26]. We now consider the prefrontal regions which has specifically evolved in primates and humans, endowing them with additional adaptive flexibility.

### Contextual models and the mid-lateral prefrontal cortex

The mid-lateral PFC (midlPFC) mainly corresponds to BA9 and BA46 located in front of the clPFC. The midlPFC is mainly connected to the clPFC, the laOFC, vmPFC, the anterior dmPFC and the frontopolar cortex (see below) [95, 130–134]. As indicated above, the clPFC enables to select selective models within the actor task set according to contextual cues associated with selective models. In contrast, there is ample empirical evidence from monkey electrophysiological recordings, human neuroimaging and lesion studies showing that the midlPFC is involved in proactively eliciting and maintaining actor task sets in relation with the occurrence of temporally-distant cues, a notion we previously referred to as episodic control (e.g., [81, 87, 135–144]). In human neuroimaging experiments, furthermore, effective connectivity analyses provide evidence that the midlPFC operates from these cues in a top-down fashion onto clPFC for proactively eliciting and maintaining actor selective models to guide behavior [87, 141, 142]. The midlPFC also appears to similarly operate onto the vmPFC in relation with actor predictive models. The midlPFC was indeed activated when the reward values of A–O associations are proactively recomputed according to contextual cues [145].

Overall, these findings support the idea that the midlPFC encodes the notion of contextual models we defined above as learning contextual cues predicting task set reliability. Contextual models index task sets through external cues that act as proactive predictors of their reliability. The occurrence of such cues enables the brain to proactively update actor task set reliability monitored in the vmPFC contributing to maintain or inhibiting the current actor task set. This further allows proactively building a new context-dependent task set from long-term memory during the process of task set creation. In humans, contextual models may further allow for updating the reliability of counterfactual task sets monitored in the frontopolar cortex (see below).

### Monitoring counterfactual task sets and the frontopolar cortex

The PFC has further evolved in humans in its most rostral portion with the emergence of a lateral frontopolar region (fpPFC) [146, 147], which apparently has no homologs in monkeys [148, 149]. Collins & Koechlin [34] demonstrated behaviorally that in uncertain and non-stationary and open-ended environments, human adaptive behavior derives from the ability to concurrently monitor the reliability of three/four task sets, namely the actor along with two/three counterfactual task sets that do not contribute to behavior. As explained above, this notion of counterfactual task sets is critical for more efficiently regulating the creation of new task sets and consequently approximating more closely optimal adaptive processes in open-ended environments [34]. There is converging evidence from human neuroimaging studies that the fpPFC is involved in monitoring counterfactual task sets. For instance, the fpPFC is engaged in “cognitive branching”, when subjects temporarily hold on the execution of one task for performing another task in response to unpredictable events [150–153]. Furthermore, the fpPFC is involved in monitoring the opportunity to switch back and forth between two alternative courses of action [104, 154]. More recent neuroimaging results even provide direct evidence that the fpPFC monitors the reliability of two concurrent counterfactual task sets, while the vmPFC monitors in parallel the actor task set [110]. Additionally, the lateral PFC is then engaged, when one counterfactual task set becomes reliable and is retrieved as actor for guiding behavior [110]. In humans accordingly, the capacity of the monitoring buffer appears to have increased from the actor in vmPFC to counterfactual task sets in fpPFC[R1.8].

## Future research directions

### Focusing on how stimuli, actions, and outcomes are linked

In this review/perspective, we propose that in order to identify computational components of adaptive behavior and their underlying neural mechanisms, we need to examine what learning in a given task entails in terms of associating stimuli, actions, and outcomes. This allows us to distinguish between learning processes that rely on simple model-free associations (S-Rew, A-Rew, and S–A associations) versus those requiring an internal model of how stimuli, actions, and outcomes are linked in the environment (e.g., S–O and (S–)A–O associations). This distinction is critical because these different types of processes provide very different levels of adaptability for tackling learning challenges in natural environments. More importantly, these processes rely on different types of synaptic plasticity and learning mechanisms, only some of which are available in certain brain areas or regions. Distinguishing between different types of stimulus, action, and outcome associations further enables disambiguating the contributions of specific brain areas, especially within the PFC to adaptive behavior. Critically, our framework suggests specific types of interactions between multiple systems that can be tested in future experiments.

By considering and examining alternative ways that stimuli, actions, and outcomes are linked through leaning processes, one can also pinpoint how inflexibility in learning and choice emerge and what their underlying neural mechanisms are. This has important implications for understanding various behavioral impairments due to neurological disorders and could resolve many disputes about the role of different cortical and sub-cortical areas in maladaptive behavior. For example, impairment in estimating action values (e.g., in the dmPFC and the striatum)––which rely on no predictive models and thus is less flexible––has a very different impact on adaptive behavior than impairment in predictive models predicting outcomes based on the same actions (e.g., in the vmPFC).

In addition, we highlighted shortcomings of most commonly used experimental paradigms to study different aspects of adaptive behavior. More specifically, one-dimensional experimental paradigms––that is, those involving only one reward attribute such as reward probability or reward magnitude––cannot distinguish between different types of learning processes. To carefully examine flexibility in learning and choice behavior, one needs to consider tasks with multiple types of reward information. Only when multiple types of reward information are present, can one tease apart different mechanisms of adaptability and their neural substrates. Nonetheless, distinguishing between these mechanisms also requires utilizing computational models that incorporate multiple components/systems for capturing different types of learning or associations between stimuli, actions, and outcomes. For example, ignoring how reward probability and magnitude are combined can result in erroneous conclusions about the impacts of volatility on learning and choice behavior.

Inference is an important component of predictive and contextual models as well as their interactions with other internal models. However, we would like to emphasize that basic inference processes for estimating outcomes or for combining inferential and non-inferential processes can be approximated using very simple mechanisms [13, 15, 74, 75]. Finally, our framework highlights the diversity and heterogeneity of learning mechanisms. As described below, this has important implications for the notion of subjective values and utility maximization that are often used to account for learning and decision-making.

### Moving beyond the notion of common currency and utility maximization

As mentioned above, to tackle the challenges of adaptive behavior in uncertain, ever changing, and open-ended environments, the brain has to rely on dynamic interaction between multiple systems that aim to link stimuli, actions, and outcomes in different fashions. This means the amount by which each system contributes to behavior constantly changes over time. As described earlier in details, there are many representations of values (S-Rew, A-Rew, current subjective value anticipation based on predictive models, cached values in selective models, etc.) that all dynamically compete for action selection with different weights depending on internal and external factors. As a result, the present framework proposes that there is no integration of all these value representations into a unique utility function or equivalently a common currency to be maximized as often postulated [99], even if the result of these various competitive processes may appear in some situations as-if the animal maximizes a unique subjective utility function. More importantly, even though this “as-if” approach could explain some aspects of choice behavior, it may not be the best approach to understand how different brain regions/systems concurrently contribute to adaptive behavior and might account for the various well-known discrepancies between human decision-making and utility maximization [155]. Consistent with this view, the notion of utility function and utility maximization have been developed to formalize the normative concept of rational choice rather than any decision-making processes. This formalization/axiomatization further applies to what Savage [156] describes as “small worlds”, i.e., stationary, closed, risky but fully known environments with stable agents, in sharp contrast to the uncertain, changing and open-ended environments that animals with changing internal states face in real-life.

Indeed, there is very little evidence for the fusion of different reward attributes into a single utility quantity even under the oversimplified condition in which choice options consist of only two attributes, reward probability and magnitude [11, 13, 80, 157]. Only a handful of studies have actually tested these two alternative possibilities to examine whether different reward attributes are fused into a utility quantity before making decisions or not. Additionally, there is evidence that the properties of the animal’s environment determine what learning systems should impact behavior more strongly and that these impacts change over time depending on the reliability of these systems [11, 13, 21, 23, 79].

More generally, the present framework suggests that there is no overarching system computing and maximizing a unique utility quantity as well as controlling and weighting the concurrent contribution of each system to behavior. Although within the actor task set, different systems/models are assumed to influence behavior with different weights, this weighting implies no superordinate controllers. Instead, the weighting relies on the reliability of internal models that within the actor task set, directly reflects the gain by which neural representations and signals dynamically encode internal models and consequently regulate their influence on behavioral choices.

Accordingly, the notion of utility function and utility maximization might provide limited insights into understanding computations involved in adaptive behavior and their neural substrates in the PFC. We hope the present framework will help to move forward and better understand the PFC function and how the multiple neural systems and internal models linking stimuli, actions, outcomes and rewards along with their dynamic interactions determine learning and behavioral choices.
